# Augmented Fixation of Distal Clavicle Fractures: High Union, Moderate Complications

**DOI:** 10.7759/cureus.117352

**Published:** 2026-10-02

**Authors:** Imran Khan, Tahreem Fatima, Kashif Memon, Muhammad Saleem Khan

**Affiliations:** 1 Trauma and Orthopaedics, Queen Elizabeth Hospital Birmingham, Birmingham, GBR

**Keywords:** arthrex tightrope, distal clavicle fracture, open reduction internal fixation, shoulder trauma surgeon, upper limb surgery

## Abstract

Introduction

Unstable lateral clavicle fractures (Neer type II) present significant surgical challenges. Combined fixation using a locking clavicular plate and suture-button (e.g., TightRope (Arthrex, Inc., Naples, FL)) augmentation is a biomechanically appealing technique designed to provide both anatomical stability and coracoclavicular (CC) reconstruction. This study reports the early clinical and radiological outcomes of this combined approach in a representative NHS patient cohort.

Materials and methods

A retrospective case series was conducted of 18 consecutive patients (mean age 48.5 years, 12 males). All patients received a pre-contoured locking plate (Arthrex/Synthes) combined with a suture-button construct for CC augmentation (knotless/knotted TightRope, FiberTape (Arthrex, Inc., Naples, FL) or EndoButton (Smith+Nephew, Watford, UK)). Primary outcomes were radiographic union and complications. Secondary outcomes included range of motion (ROM) and patient-reported outcome measures (PROMs) (Visual Analogue Scale (VAS), QuickDASH, Oxford Shoulder Score).

Results

Among the 18 patients with documented radiological follow-up, the mean time to union was 23.9 weeks (range 17.4-34.8 weeks). At a mean follow-up of 13.2 months, functional ROM was achieved in 15 patients (84%). Preliminary PROMs from a subset of patients (n = 10) showed a mean Visual Analogue Scale (VAS) pain score of 1.1, mean QuickDASH of 20.1 and mean Oxford Shoulder Score of 52.5. The overall complication rate was 16.7% (3/18), with one patient experiencing two complications. Complications included one implant failure (TightRope construct rupture) requiring revision surgery, one deep wound infection requiring debridement and implant removal, one case of frozen shoulder and one instance of transient neurological symptoms. No cases of plate failure or non-union were observed, although two patients (11.1%) required re-operation (implant removal for infection in one and a planned secondary ligament reconstruction for the failed TightRope construct in the other).

Conclusion and significance

Combined locking plate and suture-button augmentation for unstable lateral clavicle fractures results in reliable bony union and good early functional outcomes in an NHS setting. While the complication rate reflects the complexity of these injuries and the learning curve associated with the technique, the absence of catastrophic implant failure or non-union is encouraging. This dual-construct technique offers a viable and effective alternative to traditional hook plate fixation.

## Introduction

The clavicle is an essential strut for shoulder function, connecting the upper limb to the axial skeleton. Fractures of the lateral (distal) third account for approximately 10-30% of all clavicle fractures and present distinct management challenges [1,2]. Due to the proximity of the fracture to the acromioclavicular (AC) joint and the coracoclavicular (CC) ligaments, these fractures are inherently unstable, especially in Neer type IIb patterns where the CC ligaments are detached from the medial fragment. This instability results in high non-union rates, reported to be as high as 30-44% with non-operative management [3].

Surgical intervention is therefore often indicated for displaced, unstable lateral-end fractures. Traditional techniques, such as hook plate fixation or locking plate alone, have demonstrated efficacy but are associated with notable drawbacks. Hook plates, while providing stable fixation, frequently cause subacromial impingement, acromial osteolysis and often necessitate a second surgery for implant removal [4]. Locking plate fixation alone may provide insufficient vertical stability in the absence of intact CC ligaments, leading to a risk of fixation failure or delayed union [5].

To address these limitations, contemporary surgical strategies combine rigid bony fixation with a locking plate and ligamentous augmentation of the AC/CC complex. Suture-button devices, such as the TightRope system (Arthrex, Inc., Naples, FL), act as an internal splint, restoring vertical stability and neutralising deforming forces across the fracture [6]. This combined construct aims to create a biomechanically superior environment for bone healing, allowing for early mobilisation and reducing complications.

A recent meta-analysis by Memon et al. has reinforced this approach, demonstrating that augmented fixation results in significantly higher functional outcome scores (Constant-Murley Score), faster time to union, lower pain scores and fewer overall complications compared to non-augmented techniques [7].

That meta-analysis pooled existing trial and cohort data; what it could not capture is whether those results hold up in unselected, real-world NHS practice, where implant choice, surgeon experience and follow-up documentation vary. This is the specific gap this study addresses: a single-centre, consecutive-patient case series describing union, complications and patient-reported outcome measures (PROMs) under routine NHS conditions. Because every patient in this cohort received augmentation by design, the study is a feasibility and real-world safety description of the augmented technique, not a comparative test of augmentation's incremental benefit over locking plate fixation alone - that question requires a controlled comparator study and is not one this design can answer.

The purpose of this study is to present a single-centre case series of patients treated with locking plate fixation and AC joint augmentation for lateral‑end clavicle fractures, contributing real-world clinical data to the existing evidence base. As a retrospective, single-arm case series, this study is intended to describe outcomes and feasibility rather than to establish comparative efficacy.

## Materials and methods

Study design and patient selection

This was a retrospective case series of all patients who underwent surgical fixation for a lateral-end clavicle fracture at a tertiary care centre, Queen Elizabeth Hospital Birmingham, Birmingham, UK, between December 2022 and October 2025. Patients were identified from an institutional trauma database. Inclusion criteria were (1) acute lateral-end clavicle fracture, (2) treatment with open reduction and internal fixation (ORIF) using a locking clavicle plate and (3) concomitant AC joint augmentation using a suture-button device (e.g., TightRope, EndoButton (Smith+Nephew, Watford, UK)), suture-based cerclage or coracoid anchor. There were no exclusion criteria to capture the full spectrum of real-world practice. As the project constituted a retrospective audit of anonymised clinical data, formal research ethics review was confirmed as unnecessary by the Clinical Audit and Effectiveness Team at University Hospitals Birmingham NHS Foundation Trust, Birmingham, UK, in line with the trust's policy on service evaluation and audit. The project was registered under the Trust's Clinical Audit Registration and Management System (reference CARMS-24270).

Because concomitant AC joint augmentation was itself an inclusion criterion, this cohort cannot describe differential indications for augmenting versus not augmenting a given fracture nor compare augmented against non-augmented fixation. In practice, surgeons based the decision to augment on Neer subtype and the degree of CC ligament disruption seen intraoperatively.

Data collection and outcomes

Data were extracted from electronic patient records and included: patient demographics (age, sex), laterality, mechanism of injury (MOI), date of surgery (DOS) and operating surgeon. The primary outcome measures were the following.

Radiological Union

Time to union (weeks) and non-union rate were documented in follow-up radiology reports. Union was determined by the treating surgeon based on radiographic assessment of bridging callus and absence of a visible fracture line on plain radiographs. Radiographs were not independently reviewed by a second assessor, and no standardised radiographic union scoring system was applied.

Functional Outcomes

Range of motion (ROM) was assessed at six weeks, three months, six months and one year. PROMs recorded at the latest follow-up included the VAS for pain (0-10), the QuickDASH Score (0-100, higher scores indicating greater disability) [8] and the Oxford Shoulder Score (OSS) (12-60, higher scores indicating better function) [9]. Permission for the use of OSS was obtained from the concerned authority.

Complications

All intra-operative and post-operative complications were recorded, including infection, implant failure, non-union, neurological symptoms and the need for re-operation.

Smoking Status

Smoking status was recorded as a potential confounder for bone healing.

Surgical technique

All procedures were performed using a general technique involving open reduction of the lateral-end fracture through a deltopectoral or strap incision, followed by fixation with a pre-contoured locking distal clavicle plate (predominantly Arthrex or Synthes). AC joint augmentation was performed using various suture-button constructs, most commonly an Arthrex TightRope (knotted or knotless), which was passed through the coracoid process and secured to the plate or clavicle (Figure 1). In one case, a non-suture-button augmentation with FiberTape (Arthrex, Inc., Naples, FL) and a coracoid anchor was used.

**Figure 1:**
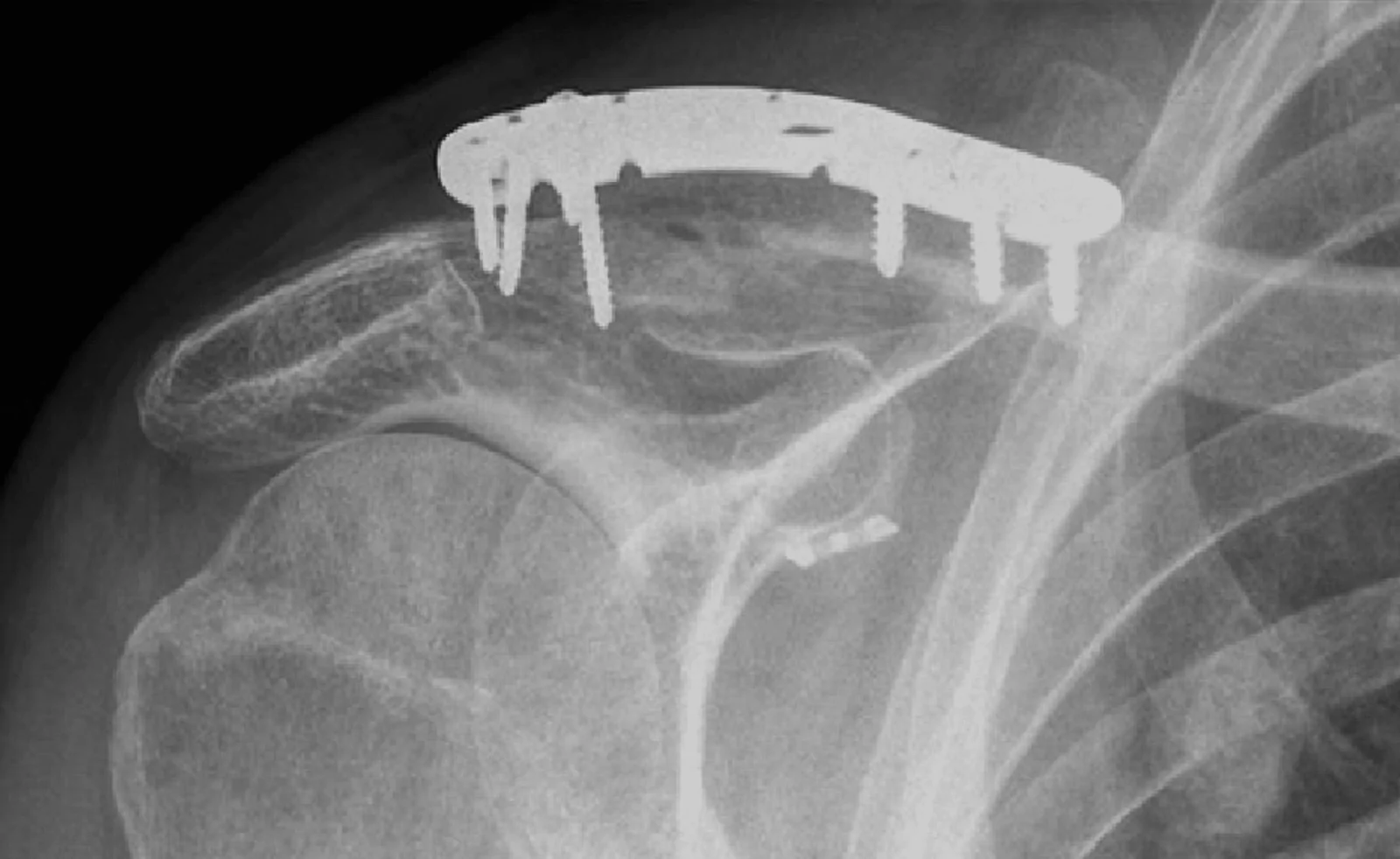
Postoperative anteroposterior radiograph showing fixation with an Arthrex distal clavicle locking plate and TightRope coracoclavicular augmentation, with satisfactory fracture reduction and implant position.

Statistical analysis

Data were collated and analysed in Microsoft Excel (Microsoft® Corp., Redmond, WA). As this is a descriptive, single-arm case series without a comparator group, only descriptive statistics were used: means with standard deviations and ranges for continuous variables and frequencies with percentages for categorical variables. No inferential tests were performed. Normality was therefore not formally assessed, since no analysis assumed a particular distribution.

## Results

Patient demographics

A total of 18 patients met the inclusion criteria. The cohort comprised 12 males (66.7%) and six females (33.3%), with a mean age of 47.7 years (range 24-87 years). The right side was affected in 10 patients (55.6%) and the left in eight patients (44.4%). The most common MOI was a fall from height in 11 patients (61.1%), followed by road traffic collisions in three patients (16.7%). A summary of patient demographics is provided in Table 1.

**Table 1:** Patient demographics and clinical characteristics.

Characteristic	Value (n = 18)
Age, years - mean ± SD (range)	47.7 ± 17.9 (24-87)
Sex, male	12 (66.7%)
Sex, female	6 (33.3%)
Laterality, right	10 (55.6%)
Laterality, left	8 (44.4%)
Mechanism of injury, fall from height	11 (61.1%)
Mechanism of injury, road traffic collision	3 (16.7%)
Mechanism of injury, road traffic accident	1 (5.6%)
Mechanism of injury, assault	1 (5.6%)
Mechanism of injury, polytrauma	1 (5.6%)
Mechanism of injury, skiing accident	1 (5.6%)
Smoking status, non-smoker	7 (38.9%)
Smoking status, smoker	1 (5.6%)
Smoking status, not documented	10 (55.6%)

Radiological outcomes

Radiological union data were available for 18 patients. Bony union was achieved in all 18 patients, resulting in a union rate of 100% at final follow-up (Figure 2). One patient with early screw pull-out subsequently progressed to union without re-operation. The mean time to union was 23.9 weeks (range 17.4-34.8 weeks) for the seven patients in whom this was precisely documented. No persistent non-unions were observed.

**Figure 2:**
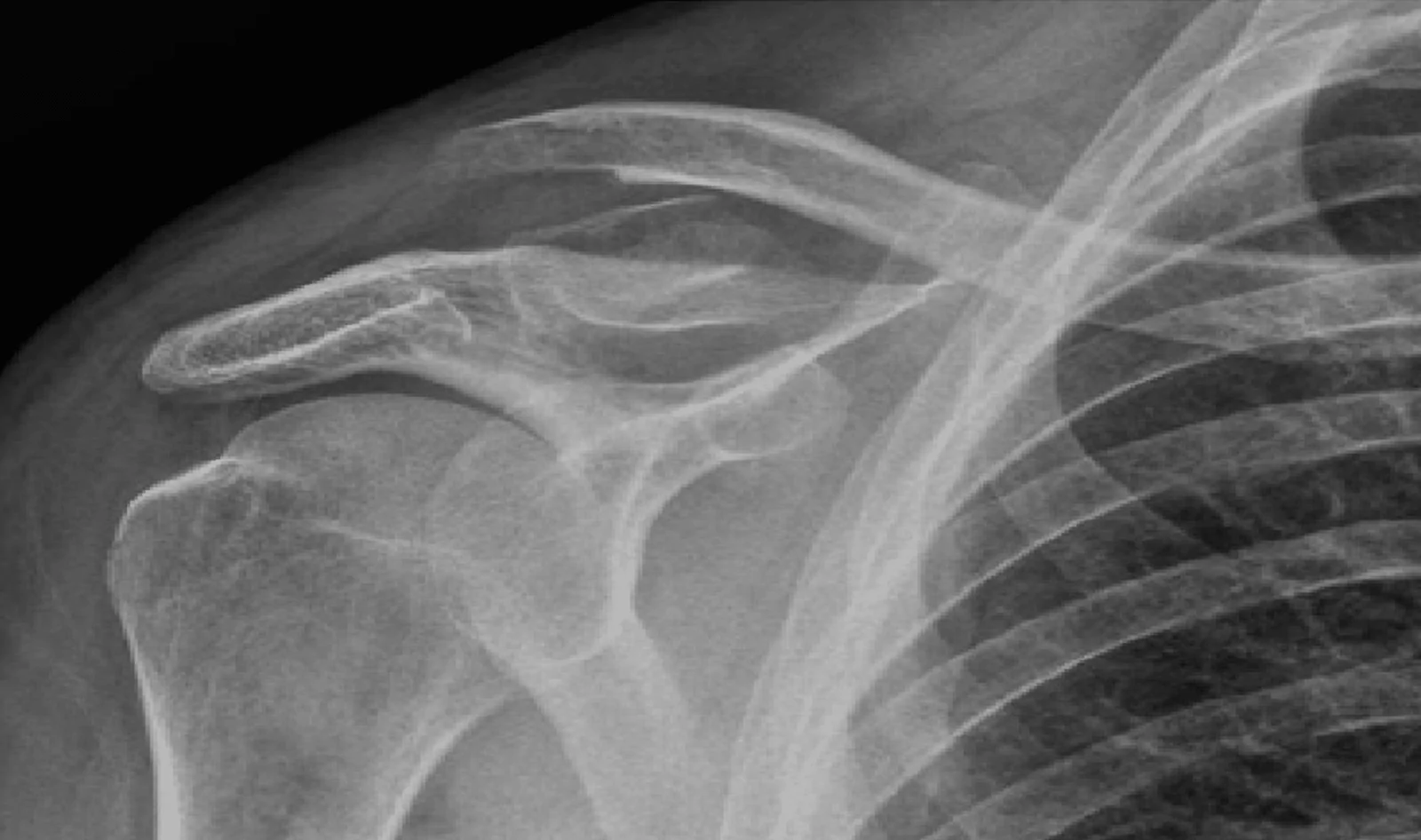
Preoperative anteroposterior radiograph demonstrating a displaced right distal clavicle fracture in a 49-year-old male patient.

Functional outcomes

Functional outcomes were assessed through ROM recordings and PROMs. ROM at various time points demonstrated progressive recovery, with most patients achieving full or near-full ROM by six months to one year. PROMs were available for 10 of the 18 patients. At the final follow-up (mean 13.2 months), the mean VAS pain score was 1.1 (range 0-7). The mean QuickDASH Score was 20.1 (range 0-63.6), and the mean OSS was 52.5 (range 35-60). These scores indicate a good level of post-operative function with minimal pain. A summary of clinical outcomes is presented in Table 2.

**Table 2:** Summary of clinical outcomes.

Outcome measure	n	Mean ± SD (range)
Mean time to union, weeks	6	23.9 ± 6.0 (17.4-34.8)
VAS Pain Score, 0-10	7	1.1 ± 1.7 (0-4)
QuickDASH Score, 0-100	12	20.1 ± 21.1 (0-63.6)
Oxford Shoulder Score, 12-60	12	52.5 ± 9.1 (35-60)

Complications

Complications were observed in three of the 18 patients (16.7%), with one patient experiencing two complications.

Implant-Related

One patient experienced a failed TightRope construct requiring revision surgery (LARS procedure advised). One patient had early screw pull-out but progressed to union without re-operation.

Infection

One patient developed a deep post-operative wound infection requiring surgical debridement and subsequent implant removal.

Secondary Frozen Shoulder

One patient developed a secondary frozen shoulder post‑operatively, which responded to physiotherapy.

Re-operation

Two patients (11.1%) required a re-operation: one for debridement and implant removal due to infection, and one was advised to have a LARS procedure for a failed TightRope.

Intra-operative

No intra-operative complications were documented in 17 out of 18 cases. One patient had an intra-operative difficulty where a planned knotless TightRope procedure was abandoned and converted to a knotted TightRope due to difficulty with coracoid drilling/button passage.

## Discussion

This single-centre case series suggests that locking plate fixation with AC joint augmentation is a reliable and effective treatment for lateral-end clavicle fractures. Our findings align closely with the recent meta-analysis by Memon et al., which reported high union rates (97-100%) and improved functional outcomes with augmented constructs [7]. The 100% union rate at final follow-up in our series (including one patient who healed after initial screw pull-out) supports the durability of this technique in achieving bony consolidation, even when early implant issues occur.

The mean time to union in our series (23.9 weeks) was longer than the 13.9 weeks reported in the pooled analysis by Memon et al. This discrepancy is likely due to variability in follow-up schedules and the conservative documentation of union in our retrospective cohort. Many patients (11/18) did not have a precisely documented union time, and formal radiological reporting often lags actual healing. Nevertheless, all patients with complete radiological follow-up ultimately united, reinforcing the mechanical stability provided by the augmented construct.

Functional recovery in our cohort was satisfactory, with patients reporting low pain levels (mean VAS 1.1) and good shoulder function at final follow-up (mean QuickDASH 20.1, mean OSS 52.5). These PROMs suggest that patients can return to a high level of function with minimal disability [10]. One patient with a high QuickDASH Score (63.6) was noted to have "good ROM" on examination, highlighting that patient-perceived disability may differ from clinical findings. The OSS, being a joint-specific measure, provides a more holistic patient-centred assessment.

The complication rate of 16.7% is comparable to the lower range reported in the literature for augmented fixation (5-18% in the Memon meta-analysis) and may compare favourably to previously reported hook plate series, although the absence of a concurrent comparator group in this study limits direct comparison [7]. The failed TightRope highlights a device-specific mode of failure that surgeons should counsel patients about. The case of sensory loss is a reminder of supraclavicular nerve risk during surgical approach - a complication often under-reported but potentially preventable with careful dissection.

Our findings are broadly consistent with the wider comparative literature on distal clavicle fracture fixation. Salazar et al. found comparable union and complication rates between locking plate fixation performed with and without CC ligament augmentation and questioned whether augmentation is necessary in all cases [11]; our series, in which augmentation was used uniformly, cannot address this question directly, but the absence of plate failure or non-union supports augmentation as a safe adjunct even if its incremental benefit over locking plate fixation alone remains debated. Hohmann et al. reported on TightRope augmentation of Neer type II fractures using distal radius locking plates and likewise achieved reliable union with acceptable complication rates, supporting the general principle of combining rigid plate fixation with a dynamic ligamentous construct [12]. Cho et al. described a suture-button technique for Neer type IIB fractures with low complication rates and good functional recovery, echoing our results with the TightRope construct specifically [13]. Most recently, Kim and Park compared locking plate fixation (with or without cerclage augmentation) against traditional hook plates and found that the locking plate group avoided the subacromial impingement and routine implant-removal surgery characteristic of hook plates, while achieving comparable union [14]. This mirrors the rationale underlying our own move away from hook plate fixation and reinforces that augmented locking plate constructs represent a viable alternative for this fracture pattern across different healthcare settings and implant choices.

This study's principal strengths are its inclusion of all consecutive eligible patients, which limits selection bias, and its complete reporting of treatment failures and re-operations.

This study has several limitations inherent to its retrospective, single-centre design. The sample size is relatively small, limiting statistical power for subgroup analysis. Data collection depended on clinical record accuracy, leading to missing data points (e.g., exact time to union). The variety of implants and slight variations in surgical technique across five different surgeons, while reflecting real-world practice, introduce heterogeneity. Finally, PROMs were not collected pre-operatively, so the magnitude of improvement cannot be precisely quantified. Despite these limitations, the 100% union rate is encouraging. This single-arm design cannot show whether augmentation adds benefit over locking plate fixation alone, and no treatment recommendation beyond feasibility and safety should be drawn from these data.

## Conclusions

This case series shows that locking plate fixation with AC joint augmentation is feasible and safe for managing lateral-end clavicle fractures in an NHS setting. The technique achieved a high rate of bony union and, on the measures reported here, an acceptable complication profile. Whether augmentation adds benefit over locking plate fixation alone cannot be determined from this uncontrolled design, and a comparative study is needed before it can be recommended as a primary treatment rather than a feasible option. A prospective, comparative study against non-augmented locking plate fixation is needed, using a standardised radiographic union score and PROMs collected before surgery as well as after.
